# Prognostic Factors and a Nomogram Predicting Overall Survival in Patients with Limb Chondrosarcomas: A Population-Based Study

**DOI:** 10.1155/2021/4510423

**Published:** 2021-05-15

**Authors:** Xinjie Wu, Yanlei Wang, Wei Sun, Mingsheng Tan

**Affiliations:** ^1^Peking University China-Japan Friendship School of Clinical Medicine, Beijing 100029, China; ^2^Department of Orthopedic Surgery, China-Japan Friendship Hospital, Beijing 100029, China; ^3^Beijing University of Chinese Medicine, Beijing 100029, China

## Abstract

**Introduction:**

We aimed to develop and validate a nomogram for predicting the overall survival of patients with limb chondrosarcomas.

**Methods:**

The Surveillance, Epidemiology, and End Results (SEER) program database was used to identify patients diagnosed with chondrosarcomas, from which data was extracted from 18 registries in the United States between 1973 and 2016. A total of 813 patients were selected from the database. Univariate and multivariate analyses were performed using Cox proportional hazards regression models on the training group to identify independent prognostic factors and construct a nomogram to predict the 3- and 5-year survival probability of patients with limb chondrosarcomas. The predictive values were compared using concordance indexes (*C*-indexes) and calibration plots.

**Results:**

All 813 patients were randomly divided into a training group (*n* = 572) and a validation group (*n* = 241). After univariate and multivariate Cox regression, a nomogram was constructed based on a new model containing the predictive variables of age, site, grade, tumor size, histology, stage, and use of surgery, radiotherapy, or chemotherapy. The prediction model provided excellent *C*-indexes (0.86 and 0.77 in the training and validation groups, respectively). The good discrimination and calibration of the nomograms were demonstrated for both the training and validation groups.

**Conclusions:**

The nomograms precisely and individually predict the overall survival of patients with limb chondrosarcomas and could assist personalized prognostic evaluation and individualized clinical decision-making.

## 1. Introduction

Chondrosarcoma is the most common bone sarcoma in aging populations and accounts for 30% of all malignant bone tumors [1, 2]. Central chondrosarcoma is the most common type and accounts for 70% of all chondrosarcomas that involve the proximal femur and proximal humerus [3]. It is generally thought that, because of their extracellular matrix, low percentage of dividing cells, and poor vascularity, chondrosarcomas are chemo- and radiotherapy-resistant. Surgical resection is the most widely accepted treatment modality for chondrosarcoma. For selected patients, however, surgery alone is clearly an inadequate treatment [4, 5]. Rare histologic chondrosarcoma subtypes, many with characteristic clinicopathologic features, share the challenges for care of the common subtypes. The disease demands concerted effort from researchers to undertake collaborative research if outcomes are to improve.

Survival is multifactorial; therefore, no single factor can accurately predict survival outcomes for patients with limb chondrosarcoma. Thus, it would be desirable to establish a statistical prediction model that could integrate all individual prognostic factors to precisely predict the survival of limb chondrosarcoma patients. Nomograms are reliable and convenient statistical tools used to predict the overall probability of a specific outcome in various diseases by incorporating numerous prognostic factors [6, 7]. However, to the best of our knowledge, no previous research has reported a comprehensive nomogram that predicts the overall survival of patients with limb chondrosarcoma. We therefore aim to develop and validate a nomogram for predicting the overall survival of patients with limb chondrosarcomas.

## 2. Methods

### 2.1. Data Source and Inclusion Criteria

The present study analyzed the deidentified data obtained from the SEER 18 registry via SEER∗Stat software (version 8.3.5; NCI, Bethesda, USA). The SEER Program is one of the largest registry sources of cancer information supported by the National Cancer Institute of the United States and covers approximately 30% of the U.S. population [8].

Inclusion criteria were as follows: (1) diagnosis of chondrosarcoma as primary malignancy, (2) positive histological confirmation of chondrosarcoma, (3) site limited to the limb, (4) known survival months after diagnosis and cause of death, and (5) complete follow-up. Exclusion criteria were as follows: (1) chondrosarcoma not the first cancer; (2) clear cell chondrosarcoma or mesenchymal chondrosarcoma; (3) unknown or blank information about tumor size, grade, stage, laterality, and total number of tumors; and (4) unknown use of surgery, radiotherapy, or chemotherapy.

### 2.2. Prognostic Factors

Data on patient age, sex, race, year of diagnosis, primary site, tumor size, histology, grade, total number of tumors, laterality, stage, use of surgery, use of radiation, use of chemotherapy, and survival time until death or the time of the last follow-up were collected from the SEER database. The age at diagnosis was categorized as younger than 40, 40-59, and 60 years and older [9]. Race was categorized as White, Black, and others (American Indian/Alaskan Native, Asian/Pacific Islander). Histologic subtypes were categorized as conventional, myxoid, and dedifferentiated chondrosarcoma. The pathologic grade was classified according to four categories: grades I, II, III, and IV. Tumor size was classified into three groups (<8, 8-13, and >13 cm) [10, 11]. The total number of tumors was categorized into two groups (*n* = 1 and *n* > 1). The tumor stage was categorized as localized, regional, and distant. As described in the 2018 version of the *Summary Stage Manual* provided by SEER (https://seer.cancer.gov/tools/ssm/), localized tumors were defined as a tumor confined to the periosteum. Regional tumors were defined as a tumor that has extended beyond the periosteum without distant metastasis.

### 2.3. Statistical Analysis

All statistical analyses were completed using R software (version 3.3.0; http://www.r-project.org/). Continuous variables that conformed to a normal distribution were expressed as the mean ± SD values and compared with Student's *t*-test. Categorical variables were expressed as frequencies and proportions and compared with the chi-squared and Fisher exact tests. The entire database of patients was randomly divided into a training group and a validation group to construct and validate the nomograms via the R caret package (https://cran.r-project.org/package=caret). Survival curves were created using Kaplan-Meier methods for selected variables performed for overall survival and compared with the log-rank test. Univariate and multivariate analyses were performed using Cox proportional hazards regression models to determine the factors associated with survival. Based on the predictive model with identified prognostic factors, a nomogram was constructed to predict the 3- and 5-year survival rates of patients. The nomogram was validated by a concordance index (*C*-index) and calibration curve both internally (training group) and externally (validation group). For all statistical analyses, a *p* value of <0.05 was considered significant.

## 3. Results

### 3.1. Patient Characteristics

**Figure 1:**
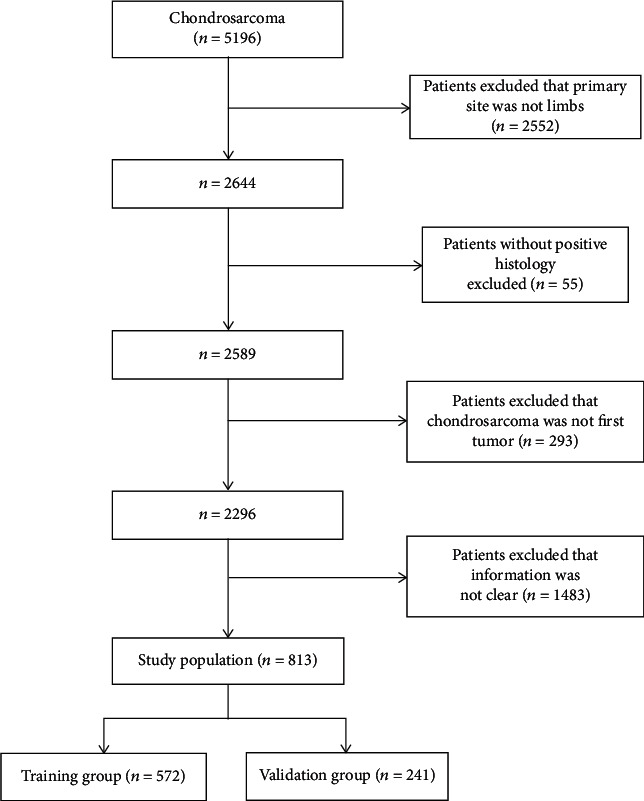
Flow diagram of the selection process in the Surveillance, Epidemiology, and End Results database.

A total of 5196 patients diagnosed with chondrosarcoma were registered in the SEER database from 1973 to 2016, of whom 2644 (50.9%) had primary tumors in the limb. According to the inclusion and exclusion criteria, 813 patients were eligible for and were eventually enrolled in the present study and randomly assigned into the training group (*n* = 572, for construction and internal validation of the nomograms) or the validation group (*n* = 241, for external validation of the nomograms) (Figure 1).

Patients characteristics are shown in Table 1. Among these 813 patients, 452 (55.6%) were male and 361 (44.4%) were female. The mean age was 51.51 years (range, 8-90 years), and the median survival time was 51 months (range, 1-155 months). Of the whole population, the majority of patients were White (*n* = 702 (86.3%)), and 668 (82.2%) had conventional chondrosarcoma. The major disease sites were the long bones of the upper and lower limb and associated joints (*n* = 281 (34.6%) and *n* = 459 (56.5%), respectively).

Grade II was the most common of the pathologic grades of chondrosarcoma (*n* = 318 (39.1%)), followed by grade I (*n* = 293 (36.0%)), grade III (*n* = 109 (13.4%)), and grade IV (*n* = 93 (11.4%)). The laterality of tumors was slightly more right than left (*n* = 419 (51.5%) and *n* = 394 (48.5%), respectively). Localized and regional were the most common tumor stages, accounting for 61.1% and 31.1%, respectively. The tumor size was less than 8 cm in 471 patients (57.9%), between 8 and 13 cm in 193 (23.7%), and more than 8 cm in 149 (18.3%). The total number of tumors equaling one accounts for 93% (*n* = 756). Moreover, surgery was performed in most patients (*n* = 771, (94.8%)). However, only a small number of patients received chemotherapy and radiotherapy (*n* = 70 (8.6%) and *n* = 48 (5.9%), respectively). Among the patients who underwent surgery, 497 of them (61.1%) had localized disease, 253 of them (31.1%) had regional disease, and 63 patients (7.7%) had distant disease. There were no significant differences for variables of patient characteristics between the training and validation groups.

### 3.2. Prognostic Factors of Overall Survival

**Figure 2:**
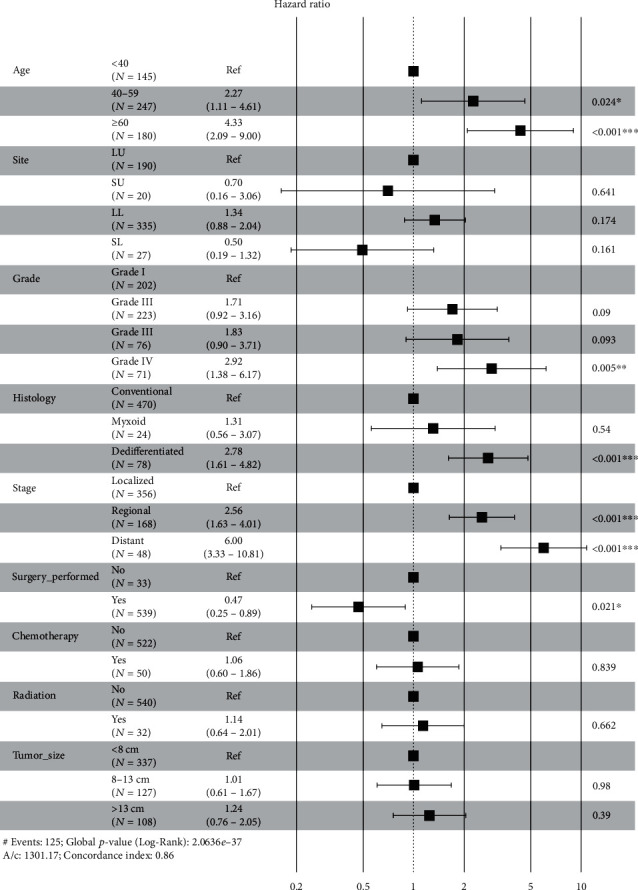
Forest plot depicting the result of multivariate analysis.

According to the univariate analysis, overall survival was significantly related to age, site, histology, grade, stage, use of surgery, radiotherapy or chemotherapy, and tumor size (Table 2). Significant factors identified by univariate analysis were further explored in multivariate analysis, which demonstrated that age, grade, stage, and use of surgery were the independent prognostic factors (Figure 2).

### 3.3. Kaplan-Meier Curve Analyses

**Figure 3:**
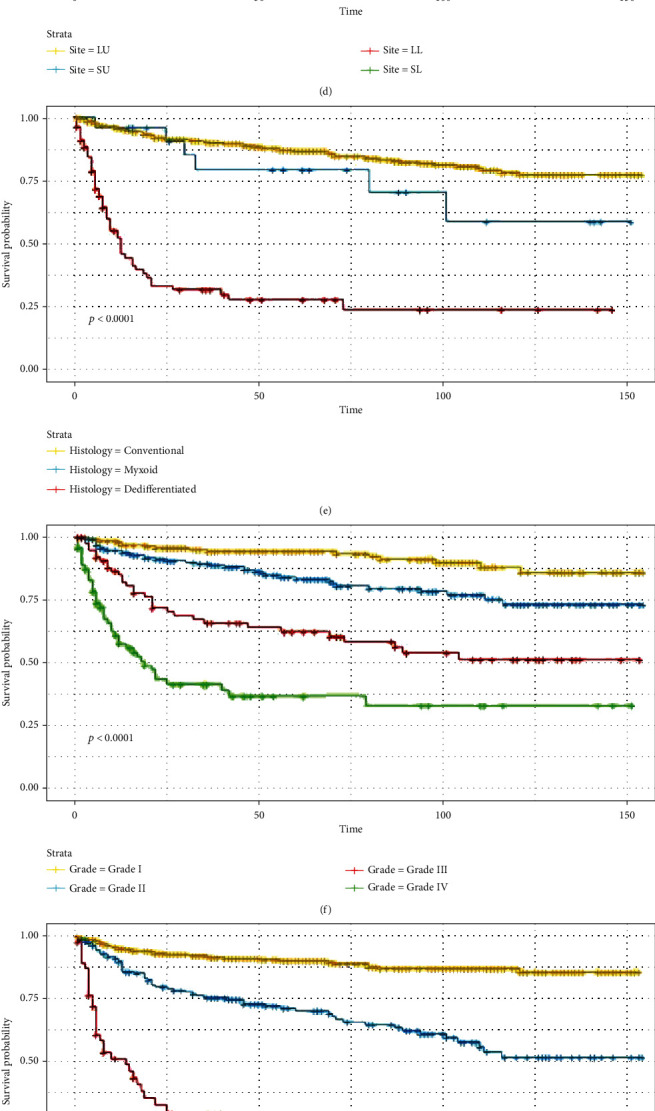
Kaplan-Meier estimated survival in patients with limb chondrosarcoma stratified by (a) age, (b) sex, (c) race, (d) site, (e) histology, (f) grade, (g) stage, (h) laterality, (i) surgery performed, and (j) total number of tumors. Note: LU: long bones: upper limb, scapula, and associated joints; SU: short bones: upper limb, scapula, and associated joints; LL: long bones of lower limb and associated joints; SL: short bones of lower limb and associated joints.

**Figure 4:**
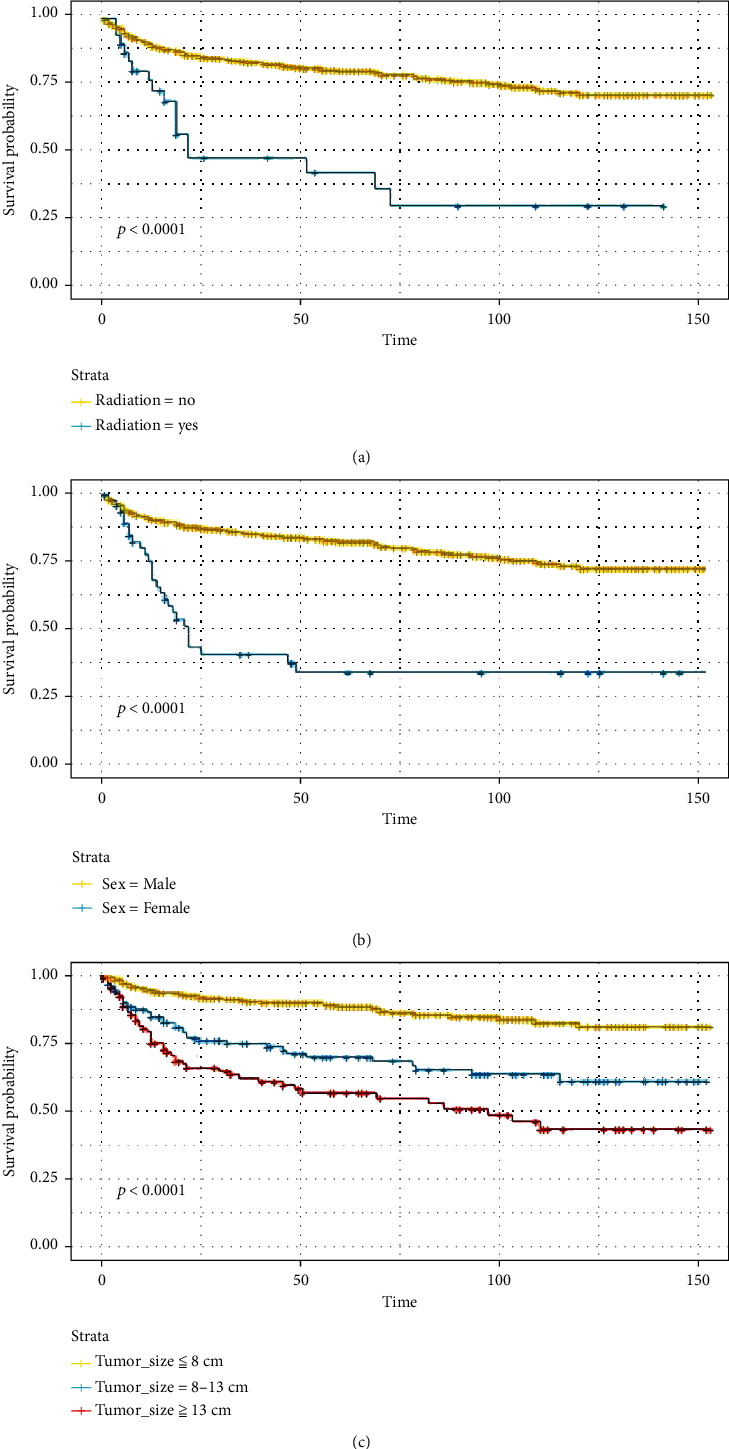
Kaplan-Meier estimated survival in patients with limb chondrosarcoma stratified by (a) radiation therapy, (b) chemotherapy, and (c) tumor size.

According to the Kaplan-Meier survival and log-rank tests, older age, long bones of lower limbs and associated joints, histologic subtype of dedifferentiated, higher grade, tumor stage of distant, no surgery, use of radiotherapy or chemotherapy, and larger tumor size resulted in worse rates of survival. However, gender, race, laterality, and total number of tumors were not significant for survival (Figures 3 and 4). Notably, the curves were crossed, suggesting that there might be multiple factors of interference.

### 3.4. Nomogram

**Figure 5:**
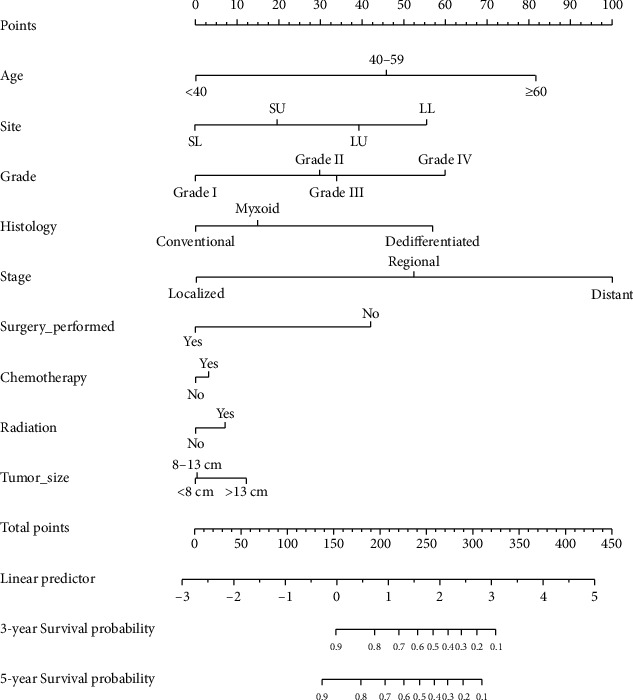
Overall survival nomogram for patients with limb chondrosarcomas. Note: LU: long bones: upper limb, scapula, and associated joints; SU: short bones: upper limb, scapula, and associated joints; LL: long bones of lower limb and associated joints; SL: short bones of lower limb and associated joints.

Based on the result of the Cox regression, we built and interpreted a nomogram for predicting the 3- and 5-year overall survival (Figure 5). The point scale at the top of each nomogram was used first to give each prognostic variable a score; then, the scale at the bottom of each nomogram was used (adding up the scores of all variables) to predict the 3- and 5-year survival probability. There were parallel lines below the figure with linear relationship scales with each other. The nomogram revealed that tumor stage “distant” contributed the highest risk to prognosis, followed by age ≥ 60 years, grade IV, long bones of lower limb and associated joints, histology subtype of dedifferentiated, no use of surgery, tumor size > 13 cm, and use of radiotherapy or chemotherapy.

**Figure 6:**
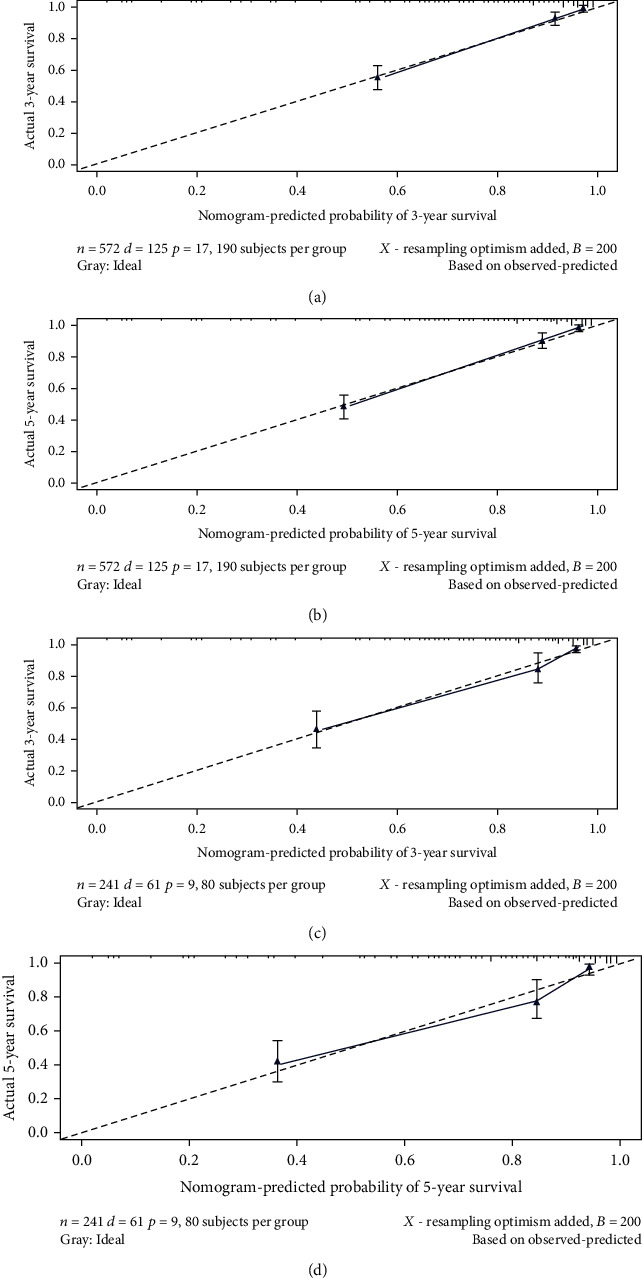
Calibration plot of the nomogram predicting overall survival in patients with limb chondrosarcoma. Internal validation of (a) actual 3-year and (b) 5-year overall survival; external validation of (c) actual 3-year and (d) 5-year overall survival. The dashed line represents an excellent match between nomogram prediction (*x*-axis) and actual survival outcome (*y*-axis). The group was divided into three groups with equal sample size for internal and external validation. Closer distances from the points to the dashed line indicate higher prediction accuracy.

The nomograms were validated internally and externally. In the training group for internal validation, the *C*-index for overall survival prediction was 0.86 (95% confidence interval (CI), 0.81-0.88). In the validation group for external validation, the *C*-index for overall survival prediction was 0.77 (95% CI, 0.73-0.79). Internal and external calibration plots for 3- and 5-year overall survival showed excellent agreement between nomogram predictions and observed outcomes (Figure 6).

## 4. Discussion

Various prognostic factors influence the survival outcome of patients with cancer, and the ability of a single prognostic factor has limited capability to predict individual survival probability. Moreover, relying merely on traditional staging systems is not enough to accurately assess the cancer prognosis [11–13]. Recently, nomograms have been widely used as reliable and convenient tools for the individualized prediction of a patient's survival outcome. However, to date, there are no studies that report nomograms that predict survival specifically for patients with limb chondrosarcomas. Therefore, utilizing the SEER database which covers approximately 28% of the overall U.S. population, we developed a comprehensive nomogram to predict 3- and 5-year overall survival for patients with limb chondrosarcoma.

The majority of included patients were older than 40 years of age, and the prognosis for survival worsened with increased age, according to the Kaplan-Meier curves and log-rank analyses. Similar results were also observed in previous studies including chondrosarcoma patients in other sites [14–16]. van Praag et al. found that patients older than 50 years of age had worse outcomes [17]. The role of gender in chondrosarcoma remains controversial [18, 19]. In the present study, the ratio of sex was approximately 1.25 to 1. However, we did not identify sex as an independent risk factor in the Cox regression analysis.

In the present study, we did not find a significant difference for the laterality of chondrosarcoma, and the ratio of left to right was 10 to 9. The primary site of chondrosarcoma was more likely to occur in the long bones of the extremities and associated joints, which accounted for 91.0%. We found that patients with chondrosarcoma in long bones had a worse prognosis. However, this should be interpreted with caution because tumors that occurred in other sites had relatively lower incidence. In addition, the Kaplan-Meier curve for the primary site of the tumor was crossed, indicating that there may be multiple factors of interference.

Pathology plays an important role in the prognosis of patients with cancer. Conventional chondrosarcoma of bone constitutes approximately 85% of all chondrosarcomas [20]. In our study, the conventional subtype accounted for 82.2% of all chondrosarcomas. Grade I chondrosarcomas are lowly cellular, with an abundant hyaline cartilage matrix, and rarely metastasize [21]. In contrast, grade III chondrosarcomas are highly cellular, with a myxoid matrix and mitoses, and with metastases developing in 70% of patients [22]. In the present study, we also found that the grade played a crucial role in the survival of patients through Cox regression and the Kaplan-Meier curve and log-rank test. Notably, grading is also subject to interobserver variability, which is worrisome due to the fact that treatment for grade I and II chondrosarcomas may differ.

In addition to conventional subtypes, other rare subtypes constituted 10-15% of all chondrosarcomas [20]. In the present study, rare subtypes accounted for approximately 17% of the chondrosarcomas. Dedifferentiated chondrosarcoma, which accounts for approximately 13% of chondrosarcoma, is diagnosed when a high-grade noncartilaginous tumor borders low-grade disease [23]. Detection of skeletal and/or lung metastases at diagnosis or within 12 to 18 months is common and associated with a dismal prognosis [24]. It was reported that the 5-year survival rate of dedifferentiated chondrosarcoma was less than 20% [25]. In our research, we also found that the dedifferentiated subtype had a worse prognosis compared to conventional and myxoid subtypes.

We demonstrated that the tumor stage was an independent prognostic factor for survival, and the localized stage was related to a significant survival benefit compared to the regional or distant stage. Previous studies reported similar results for chondrosarcoma in other sites or the whole skeletal system [9, 10, 26].

Optimal surgical treatment remains under debate, including systemic chemotherapy and radiotherapy. In line with previous studies, we found that surgery confers a significant benefit in the overall survival of patients with limb chondrosarcoma. Wide, en bloc excision was the preferred surgical treatment for intermediate- and high-grade chondrosarcoma patients. Considering that chondrosarcomas grow slowly, with a relatively low fraction of dividing cells, and radiotherapy acts on dividing cells, chondrogenic tumors are considered relatively radiotherapy-resistant [27]. Radiotherapy can be considered in two situations: after incomplete resection, aiming at maximal local control (curative), and in situations where resection is not feasible or would cause unacceptable morbidity (palliative). Chemotherapy is generally not effective in chondrosarcoma, especially in the most frequently observed conventional type and the rare clear cell variant. Previous studies reported that expression of the multidrug-resistance 1 gene, P-glycoprotein, may contribute to chemotherapy resistance in vivo and in vitro [28, 29]. In our study, we also found that chemotherapy and radiotherapy had limited effects for improving the prognosis of patients.

Based on the independent predictors of overall survival that we identified, we constructed a nomogram to predict 3- and 5-year overall survival probability for patients with limb chondrosarcoma. Our nomogram can be easily used in the clinical practice to estimate a patient's prognosis. For example, a 55-year-old man was diagnosed with grade II dedifferentiated chondrosarcoma in the long bones of his lower limb and associated joints, with tumor size of 6.0 cm, along with the use of surgery and no use of chemotherapy and radiotherapy and the stage of localized. Totaling the points for this patient, we see that he would have 187.5 points in the overall survival nomogram. The results for estimated 3-year overall survival and cancer-specific survival rates would be 80% and 75%, respectively, according to the nomogram. In our study, we also investigated the performance of the nomogram through the *C*-index and calibration plots internally and externally. They all demonstrated excellent consistency between the prediction model and actual outcomes.

### 4.1. Limitations

Notably, there are some limitations in the present study. First, the nomogram was developed based on retrospective data. It is inevitable that some patient information was unclear or blanked. Second, due to the unavailability of some variables such as local recurrence in the database, we only considered 3- and 5-year survival as endpoints. However, local recurrence at certain time points could also be evaluated as one of the endpoints. Besides, there was no clear distinction between the type of surgery performed or chemotherapy or radiotherapy regimen. Finally, the nomogram was constructed and validated via the same database. Although we randomly divided patients into the training and validation groups to validate the nomogram internally and externally, it is more reliable to validate the nomogram prospectively or using another data source.

## 5. Conclusion

With data from a large population-based group, we were the first to develop and validate a nomogram to provide individualized estimates of rates of 3- and 5-year overall survival in patients with limb chondrosarcomas. The nomograms showed a high degree of accuracy and applicability. Using the nomogram as an effective and convenient tool, clinical practitioners could make personalized survival evaluations and identify patients with a high risk of mortality.

## Figures and Tables

**Table 1:** Characteristics of patients with limb chondrosarcomas.

Variable	Training group (*N* = 572)	Validation group (*N* = 241)	Total (*N* = 813)	*p* value
*Age at diagnosis (years)*				0.10
Mean (SD)	50.87 (16.99)	53.02 (17.24)	51.51 (17.08)	
Median (Q1, Q3)	51.5 (17.0, 102.5)	45.0 (15.0, 95.0)	51.0 (16.0, 100.0)	
Min, max	10.0, 90.0	8.0, 89.0	8.0, 90.0	
*Age (years)*				0.07
<40	145 (25.3%)	55 (22.8%)	200 (24.6%)	
40-59	247 (43.2%)	88 (36.5%)	335 (41.2%)	
≥60	180 (31.5%)	98 (40.7%)	278 (34.2%)	
*Race*				0.38
White	488 (85.3%)	214 (88.8%)	702 (86.3%)	
Black	51 (8.9%)	15 (6.2%)	66 (8.1%)	
Other	33 (5.8%)	12 (5.0%)	45 (5.5%)	
*Sex*				
Male	319 (55.8%)	133 (55.2%)	452 (55.6%)	0.88
Female	253 (44.2%)	108 (44.8%)	361 (44.4%)	
*Site*				0.27
LU	190 (33.2%)	91 (37.8%)	281 (34.6%)	
SU	20 (3.5%)	10 (4.1%)	30 (3.7%)	
LL	335 (58.6%)	124 (51.5%)	459 (56.5%)	
SL	27 (4.7%)	16 (6.6%)	43 (5.3%)	
*Histology*				0.37
Conventional	470 (82.2%)	198 (82.2%)	668 (82.2%)	
Myxoid	24 (4.2%)	15 (6.2%)	39 (4.8%)	
Dedifferentiated	78 (13.6%)	28 (11.6%)	106 (13.0%)	
*Grade*				
Grade I	202 (35.3%)	91 (37.8%)	293 (36.0%)	0.59
Grade II	223 (39.0%)	95 (39.4%)	318 (39.1%)	
Grade III	76 (13.3%)	33 (13.7%)	109 (13.4%)	
Grade IV	71 (12.4%)	22 (9.1%)	93 (11.4%)	
*Laterality*				0.52
Left	273 (47.7%)	121 (50.2%)	394 (48.5%)	
Right	299 (52.3%)	120 (49.8%)	419 (51.5%)	
*Stage*				0.19
Localized	356 (62.2%)	141 (58.5%)	497 (61.1%)	
Regional	168 (29.4%)	85 (35.3%)	253 (31.1%)	
Distant	48 (8.4%)	15 (6.2%)	63 (7.7%)	
*Surgery performed*				0.23
No	33 (5.8%)	9 (3.7%)	42 (5.2%)	
Yes	539 (94.2%)	232 (96.3%)	771 (94.8%)	
*Total number of tumors*				0.08
*n* = 1	526 (92.0%)	230 (95.4%)	756 (93.0%)	
*n* > 1	46 (8.0%)	11 (4.6%)	57 (7.0%)	
*Radiation*				0.56
No	540 (94.4%)	225 (93.4%)	765 (94.1%)	
Yes	32 (5.6%)	16 (6.6%)	48 (5.9%)	
*Chemotherapy*				0.84
No	522 (91.3%)	221 (91.7%)	743 (91.4%)	
Yes	50 (8.7%)	20 (8.3%)	70 (8.6%)	
*Tumor size*				0.29
<8 cm	337 (58.9%)	134 (55.6%)	471 (57.9%)	
8-13 cm	127 (22.2%)	66 (27.4%)	193 (23.7%)	
≥13 cm	108 (18.9%)	41 (17.0%)	149 (18.3%)	
*Survival time (months)*				0.16
Mean (SD)	61.01 (46.37)	56.06 (45.03)	59.54 (46.00)	
Median (Q1, Q3)	51.5 (17.0, 102.5)	45.0 (15.0, 95.0)	51.0 (16.0, 100.0)	
Min, max	1.0, 154.0	1.0, 155.0	1.0, 155.0	

Note: LU: long bones: upper limb, scapula, and associated joints; SU: short bones: upper limb, scapula, and associated joints; LL: long bones of lower limb and associated joints; SL: short bones of lower limb and associated joints.

**Table 2:** Univariate Cox regression analysis in the training group.

Variable	HR	Univariate analysis	*p* value
		95% CI	
*Age*			
<40 years	Ref		
40-59 years	2.81	1.48-5.57	0.003
≥60 years	7.23	3.72-14.05	<0.001
*Race*			
White	Ref		
Black	0.46	0.19-1.12	0.09
Other	0.63	0.26-1.54	0.31
*Sex*			
Male	Ref		
Female	0.85	0.60-1.23	0.41
*Site*			
LU	Ref		
SU	0.73	0.18-3.06	0.67
LL	1.70	1.13-2.56	0.01
SL	0.96	0.37-2.46	0.93
*Histology*			
Conventional	Ref		
Myxoid	1.72	0.74-3.95	0.21
Dedifferentiated	9.54	6.57-13.87	<0.001
*Grade*			
Grade I	Ref		
Grade II	2.32	1.29-4.15	0.005
Grade III	5.82	3.18-10.63	<0.001
Grade IV	13.68	7.63-24.52	<0.001
*Laterality*			
Left	Ref		
Right	1.02	0.72-1.45	0.389
*Stage*			
Localized	Ref		
Regional	3.67	2.40-5.59	<0.001
Distant	14.97	9.28-24.11	<0.001
*Surgery performed*			
No	Ref		
Yes	0.46	0.26-0.82	0.008
*Total number of tumors*			
*n* = 1	Ref		
*n* > 1	0.89	0.48-1.65	0.70
*Radiation*			
No	Ref		
Yes	3.71	2.22-6.19	<0.001
*Chemotherapy*			
No	Ref		
Yes	4.31	2.80-6.62	<0.001
*Tumor size*			
<8 cm	Ref		
8-13 cm	2.72	1.75-4.23	<0.001
≥13 cm	4.43	2.91-6.73	<0.001

Note: HR: hazard ratio; CI: confidence intervals; LU: long bones: upper limb, scapula, and associated joints; SU: short bones: upper limb, scapula, and associated joints; LL: long bones of lower limb and associated joints; SL: short bones of lower limb and associated joints.

## Data Availability

The dataset generated and analyzed during the current study is available in the National Cancer Institute's Surveillance, Epidemiology, and End Results (SEER) repository.
